# Resveratrol Suppresses PAI-1 Gene Expression in a Human *In Vitro* Model of Inflamed Adipose Tissue

**DOI:** 10.1155/2013/793525

**Published:** 2013-06-02

**Authors:** Ivana Zagotta, Elitsa Y. Dimova, Jan-Bernd Funcke, Martin Wabitsch, Thomas Kietzmann, Pamela Fischer-Posovszky

**Affiliations:** ^1^Division of Pediatric Endocrinology and Diabetes, Department of Pediatrics and Adolescent Medicine, Ulm University Medical Center, Eythstraße 24, 89075 Ulm, Germany; ^2^Department of Biochemistry and Biocenter Oulu, University of Oulu, P.O.B. 3000, 90014 Oulu, Finland

## Abstract

Increased plasminogen activator inhibitor-1 (PAI-1) levels are associated with a number of pathophysiological complications; among them is obesity. Resveratrol was proposed to improve obesity-related health problems, but the effect of resveratrol on PAI-1 gene expression in obesity is not completely understood. In this study, we used SGBS adipocytes and a model of human adipose tissue inflammation to examine the effects of resveratrol on the production of PAI-1. Treatment of SGBS adipocytes with resveratrol reduced PAI-1 mRNA and protein in a time- and concentration-dependent manner. Further experiments showed that obesity-associated inflammatory conditions lead to the upregulation of PAI-1 gene expression which was antagonized by resveratrol. Although signaling via PI3K, Sirt1, AMPK, ROS, and Nrf2 appeared to play a significant role in the modulation of PAI-1 gene expression under noninflammatory conditions, those signaling components were not involved in mediating the resveratrol effects on PAI-1 production under inflammatory conditions. Instead, we demonstrate that the resveratrol effects on PAI-1 induction under inflammatory conditions were mediated via inhibition of the NF**κ**B pathway. Together, resveratrol can act as NF**κ**B inhibitor in adipocytes and thus the subsequently reduced PAI-1 expression in inflamed adipose tissue might provide a new insight towards novel treatment options of obesity.

## 1. Introduction

Obesity is becoming an increasing public health problem worldwide. The excessive accumulation of adipose tissue leads to the development of dyslipidemia, impaired glucose metabolism, hypertension, and proinflammation, processes playing an essential role in the pathogenesis of cardiovascular disease, type 2 diabetes, the metabolic syndrome, and various cancers (reviewed by [1]). Many of those obesity-related pathophysiological conditions are associated with increased plasminogen activator inhibitor-1 (PAI-1) levels [2–6]. PAI-1 is the primary, fast-acting inhibitor of both tissue-type and urokinase-type plasminogen activators and therefore controls the regulation of the fibrinolytic system in blood [7, 8]. In addition, PAI-1 is an important regulator of extracellular matrix turnover, tissue remodeling, and fibrosis [9]. PAI-1 levels can be increased in response to hypoxia [10, 11], hormones like insulin [12, 13], coagulation factors, and cytokines (discussed by [14]). More recently PAI-1 levels have been considered as one of the biomarkers used to predict obesity-associated diseases [15]. Elevated PAI-1 mRNA levels have been found in adipose tissues from obese ob/ob mice [16] and also in human obesity with higher expression levels in visceral compared to subcutaneous adipose tissue depots [17]. Thus, high plasma PAI-1 levels are a common finding in obesity in both mice and humans [18–25]. Most importantly, the obesity-induced PAI-1 increase is reversible by lifestyle intervention. Weight loss due to calorie restriction decreased plasma PAI-1 concentrations in obese individuals [26, 27]. These data imply that substances that potentially mimic calorie restriction may be used as modulators of PAI-1 levels in the treatment of obesity and obesity-related diseases.

From a number of natural compounds mimicking calorie restriction by targeting various metabolic pathways, resveratrol gained special interest. Resveratrol is a polyphenol produced by plants in response to environmental stress and found in red grape skin, peanuts, a variety of berries, and medical plants [28]. It has been suggested to act as a calorie restriction mimetic based on data from rodents. When mice and/or rats were fed a high-fat diet, resveratrol treatment improved glucose homeostasis, mitochondrial function, lipid parameters, body weight, and survival [29–39]. While the resveratrol effects are intensively studied in animal models only few clinical trials were conducted so far to study the effects of resveratrol supplementation in the context of human obesity [40] and coronary artery disease [41]; yet there exists some controversy [42] and the effect of resveratrol in obese human individuals remains to be further investigated.

Although obesity and obesity-associated diseases seem to be positively influenced by resveratrol, not much is known about the effect of resveratrol on PAI-1 in obesity. Therefore, it was the aim of this study to investigate the effects of resveratrol on the production of PAI-1 in human adipocytes and in an *in vitro* model of human adipose tissue inflammation. We found that resveratrol reduces PAI-1 levels in adipocytes especially under inflammatory conditions. Thus, our data support the concept that resveratrol can alleviate obesity-induced upregulation of PAI-1 in human adipose tissue. 

## 2. Materials and Methods

### 2.1. Reagents and Cell Culture

All biochemicals were of analytical grade and were purchased from commercial suppliers. Resveratrol, sirtinol, and LY204002 were obtained from Sigma (Deisenhofen, Germany). SC-514 was from Merck Millipore (Darmstadt, Germany). Small molecule inhibitors were diluted in DMSO which alone was also used as vehicle control. The following concentrations of resveratrol and inhibitors were used in experiments: resveratrol 10, 50, 100 *μ*M, sirtinol 10 *μ*M; LY204002 20 *μ*M, SC-514 100 *μ*M.

Simpson-Golabi-Behmel syndrome (SGBS) preadipocytes were cultured as previously described [43]. Human primary preadipocytes were prepared by collagenase digestion from subcutaneous adipose tissue of 3 healthy women using a previously described protocol [44]. Adipogenic differentiation of SGBS and human primary and SGBS preadipocytes was induced in serum-free DMEM/F12 medium supplemented with 10 *μ*g/mL iron-poor transferrin, 10 nM insulin, 200 pM thyroid hormone, and 0.1 *μ*M cortisol and for the first four days 2 *μ*M rosiglitazone, 250 *μ*M isobutylmethylxanthine, and 25 nM dexamethasone. Cells were used for experiments on day 8 of adipogenic differentiation.

THP-1 cells (ATCC, Wesel, Germany) were cultured as described earlier [45]. Differentiation into macrophages was induced by 125 ng/mL phorbol myristate acetate for 48 h. Macrophage-conditioned medium (MacCM) was collected after additional 48 h of incubation in serum-free basal medium containing 0.5% BSA and cleared by centrifugation. MacCM from 5 independently performed productions was pooled and then used for experiments.

Mouse embryonic fibroblasts (MEFs) were maintained in DMEM supplemented with 10% fetal bovine serum (Invitrogen, Karlsruhe, Germany), 1% nonessential amino acids (Invitrogen), and 0.5% antibiotics in an atmosphere of 16% O_2_, 5% CO_2_, and 97% humidity at 37°C in a cell culture incubator. Mouse embryonic fibroblasts Sirt1^+/+^ and Sirt1^−/−^ were a generous gift from Dr. Michael McBurney (Ottawa Hospital Research Institute, Canada). We obtained AMPK*α*1,2^+/+^ and AMPK*α*1,2^−/−^ MEFs [46] from Dr. Benoit Viollet (Institut Cochin, Paris, France). Nrf2 wild-type and Nrf2 knockdown MEFs were provided by Dr. Stephan Immenschuh (Hannover Medical School, Germany).

### 2.2. RNA Preparation and Quantitative Real-Time PCR

Isolation of total RNA was performed using the peqGOLD HP Total RNA kit (Peqlab, Erlangen, Germany) following the manufacturer's instructions. One *μ*g of total RNA was used for cDNA synthesis with using SuperScript II Reverse Transcriptase (Invitrogen, Darmstadt, Germany). Quantitative real-time PCR was performed with a LightCycler 2.0 (Roche Diagnostics, Mannheim, Germany) using a LightCycler FastStart DNA Master PLUS SYBR Green I kit (Roche Diagnostics, Mannheim, Germany). The quantitative real-time PCR results were normalized using hypoxanthine phosphoribosyltransferase (HPRT) as a housekeeping gene. The following primer sets were used: human PAI-1-F (5′-ACA AGT TCA ACT ATA CTG AGT TCA CCA CGC CC-3′), human PAI-1-R sequence (5′-TGA AAC TGT CTG AAC ATG TCG GTC ATT CCC-3′), human HPRT-F (5′-GAG ATG GGA GGC CAT CAC ATT GTA GCC CTC-3′), and human HPRT-R (5′-CTC CAC CAA TTA CTT TTA TGT CCC CTG TTG ACT GGT C-3′). The experiments for each data point were carried out in triplicate. The relative quantification of gene expression was determined using the ΔΔCt method [47]. In some experiments conventional RT-PCR was performed using Sp1 as a reference gene (PAI-1-F: 5′-GTC TGC TGT GCA CCA TCC CCC-3′; PAI-1-R: 5′-GAA CAG CCT GAA GAA GTG GGG C-3′, Sp1-F: 5′-ACT ACC AGT GGA TCA TCA GGG-3′; Sp1-R: 5′-CTG ACA ATG GTG CTG CTT GGA-3′). 

### 2.3. ELISA

SGBS adipocytes were treated for 48 h with 10% MacCM, 100 *μ*M resveratrol, and 100 *μ*M SC-514 alone or in combination. The ELISA was performed using the Platinum ELISA kit for human PAI-1 (eBioscience, Vienna, Austria). Absorbance was measured on a spectrophotometer using 450 nm wavelength (ELx800 Absorbance Microplate Reader, BioTek, Bad Friedrichshall, Germany).

### 2.4. Western Blot Analyses

Western blot analyses were performed as previously described [10]. In brief, 24 h after treatment with vehicle or resveratrol cell culture medium (for PAI-1) or total cell lysates were collected and 100 *μ*g of protein was subjected to SDS-PAGE and blotted onto a nitrocellulose membrane. The following primary antibodies were used: PAI-1 (polyclonal 1 : 100) (American Diagnostics, Pfungstadt, Germany), AMPK*β*1/2 (polyclonal, 1 : 1000) (Cell Signaling, Hamburg, Germany), Nrf2 (polyclonal, Nrf2 1 : 200) (Santa Cruz, Heidelberg, Germany), and Sirt1 (polyclonal, 1 : 1000) (Santa Cruz, Heidelberg, Germany). The secondary antibody was anti-rabbit immunoglobulin G (IgG)-horseradish peroxidase IgG (1 : 5000) (Biorad, Munich, Germany). The enhanced chemiluminescence (ECL) system (Amersham, Freiburg, Germany) was used for detection. Blots were quantified by using the Fiji program (NCBI). 

### 2.5. ROS Measurement

To determine ROS production, SGBS adipocytes were incubated with 2.5 *μ*M CM-H_2_DCFDA (Molecular Probes Europe BV, The Netherlands) for 30 min at 37°C. After three washes with PBS, cells were treated with 100 *μ*M H_2_O_2_ or 10% MacCM for 15 min and analyzed by flow cytometry. 

### 2.6. Preparation of Nuclear Extracts and Electrophoretic Mobility Shift Assay (EMSA)

SGBS adipocytes were treated with 100 *μ*M resveratrol, 100 *μ*M SC-514, and 10% MacCM alone or in combination. TNF*α* (10 ng/mL) was used as a positive control. Cells were collected from 6 cm dishes by scraping and centrifugation (10,000 g for 5 min at 4°C). After washing once with ice-cold PBS, cell pellets were resuspended in 200 *μ*L low-salt buffer (10 mM HEPES-KOH pH 7.9; 1.5 mM MgCl_2_; 10 mM KCl) and incubated for 10 min on ice. After addition of 12.5 *μ*L of a 10% Nonidet P-40 solution, samples were mixed vigorously for 30 s. Nuclei were collected by centrifugation and resuspended in 25 *μ*L high-salt buffer (20 mM HEPES-KOH pH 7.9; 1.5 mM MgCl_2_; 420 mM NaCl, 0.2 mM EDTA; 25% glycerol). Both buffers were supplemented with a protease-inhibitor cocktail (Sigma), 0.2 mM PMSF, 0.5 mM dithiothreitol (DTT), and 1 mM sodium-orthovanadate before use. Nuclei were incubated 15 min on ice and vortexed periodically. Nuclear extracts were obtained by centrifugation at 12,500 g for 10 min at 4°C and stored at −80°C. Protein concentration was determined with the BCA Protein Assay Reagent kit (Pierce, Rockford, IL), according to manufacturer's instructions. Single-stranded oligonucleotides were purchased from Biomers.net (Ulm, Germany): standard NF*κ*B probe: sense, 5′-AGT TGA GGG GAC TTT CCC AGG C-3′; antisense, 5′-GCC TGG GAA AGT CCC CTC AAC T-3′. The sense oligonucleotide was labeled with *γ*-^32^P-ATP (Amersham, Freiburg, Germany) using T4-polynucleotide kinase (MBI Fermentas, St. Leon-Rot, Germany). A 2-fold molar excess of unlabeled antisense oligonucleotide was annealed, and the labeled double-stranded oligonucleotide was purified with a spin column (Micro Bio-Spin P30; Bio-Rad, Munich, Germany). Binding reactions were performed for 30 min on ice in 20 *μ*L buffer (1 mM MgCl_2_, 0.5 mM EDTA, 0.5 mM DTT, 50 mM NaCl, 10 mM Tris-HCl, pH 7.5; 4% glycerol) containing 5 *μ*g nuclear extract protein, 1 *μ*g poly (dI:dC) (Sigma), and 10,000 cpm-labeled oligonucleotide.

### 2.7. Statistics

Data represents mean ± standard error of means (SEM) of 3 independent experiments unless otherwise stated. Statistics: statistical significance was evaluated using one-way analysis of variants (ANOVA) considering *P* < 0.05 as statistically significant. 

## 3. Results

### 3.1. Concentration- and Time-Dependent Downregulation of Human PAI-1 mRNA and Protein Levels by Resveratrol in SGBS Adipocytes

**Figure 1:**
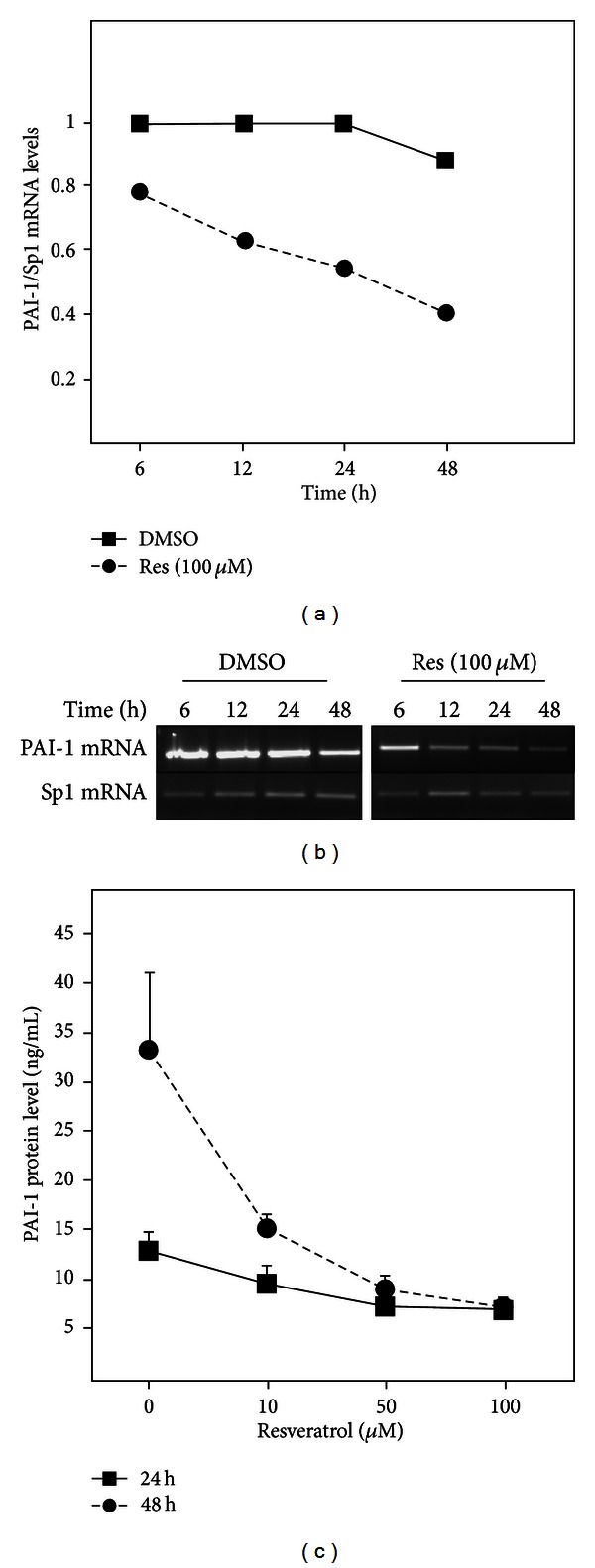
Resveratrol-dependent downregulation of PAI-1 mRNA and protein levels in SGBS adipocytes. SGBS adipocytes were incubated in adipogenic media with vehicle control (DMSO) or 100 *μ*M resveratrol (Res) for the indicated time points. (a) PAI-1 mRNA levels were measured by semiquantitative RT-PCR. Sp1 was used as a reference gene. (b) A representative RT-PCR of PAI-1 and Sp1 mRNA levels after treatment with DMSO or 100 *μ*M Res. (c) The accumulation of PAI-1 in the media was measured by ELISA after treatment with increasing doses of Res for 24 or 48 h.

To determine how resveratrol modulates PAI-1 gene expression in SGBS adipocytes, we examined PAI-1 mRNA and protein levels after treatment with increasing concentrations of resveratrol at different time points (Figure 1). Treatment of cells for 12 h, 24 h, and 48 h with different concentrations of resveratrol resulted in a reduction of PAI-1 mRNA levels in a dose-dependent manner (data not shown); 100 *μ*M resveratrol reduced PAI-1 mRNA levels by about 40% after 12 h and by about 60% after 48 h (Figures 1(a) and 1(b)). The resveratrol-mediated decrease of PAI-1 mRNA was followed by a decrease of PAI-1 protein levels. A resveratrol concentration of 50 *μ*M or 100 *μ*M diminished PAI-1 protein levels in the medium by about 50% after 24 h and by about 75% after 48 h (Figure 1(c)). Thus, resveratrol reduced PAI-1 mRNA and PAI-1 protein levels in a time- and concentration-dependent manner.

### 3.2. PAI-1 Gene Expression Is Upregulated in an *In Vitro* Model of Inflamed Human Adipose Tissue as well as in Primary Human Adipocytes

**Figure 2:**
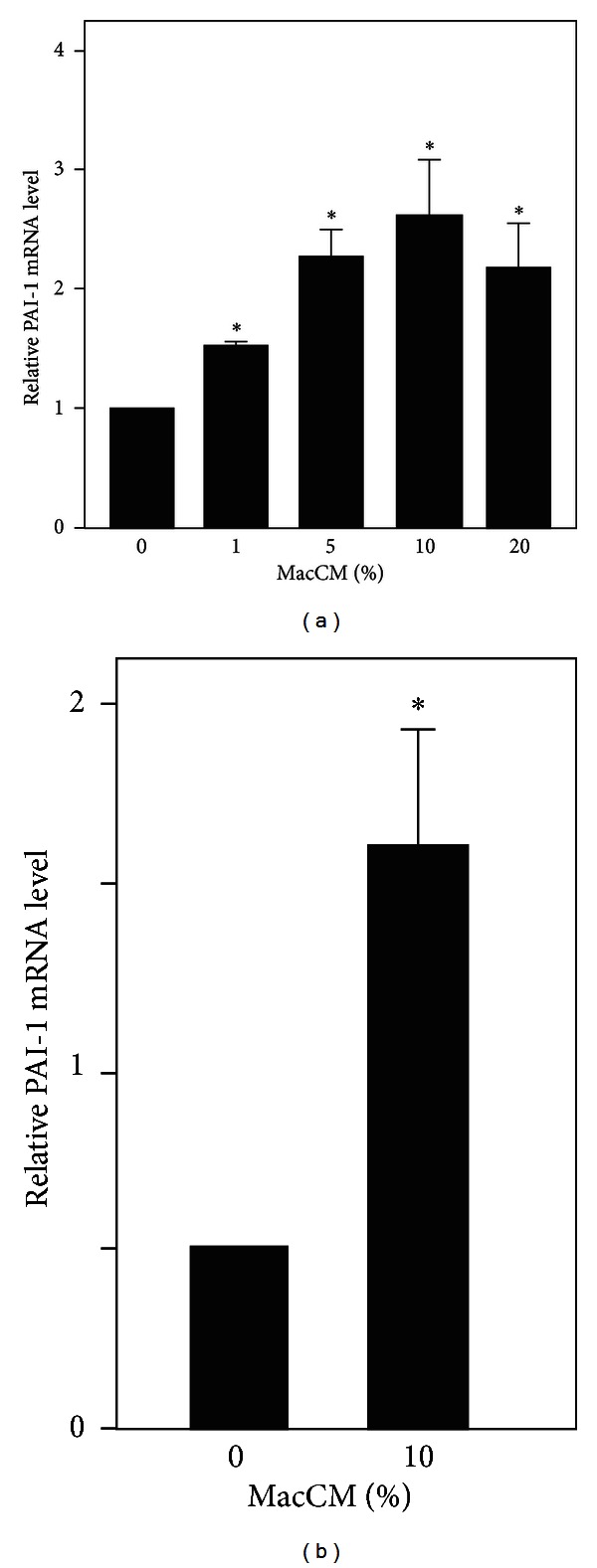
PAI-1 gene expression is upregulated in an *in vitro* model of inflamed human adipose tissue. (a) SGBS adipocytes were incubated with increasing doses of macrophage-conditioned media (MacCM) or vehicle for 48 h. PAI-1 mRNA levels were analyzed by qPCR and results were normalized to HPRT. *significant difference control versus MacCM. (b) Primary human *ex vivo* differentiated adipocytes isolated from 3 patients were treated with 10% macrophage-conditioned media (MacCM) or vehicle for 48 h. PAI-1 mRNA levels were analyzed with qPCR and normalized to HPRT. *significant difference control versus MacCM.

Obesity is associated with low-grade chronic inflammation [48] and increased circulating PAI-1 levels [6]. Therefore, we mimicked human adipose tissue inflammation by using our previously described *in vitro* model system [49] where we incubated SGBS adipocytes with medium supplemented with increasing doses of macrophage-conditioned medium (MacCM) for 48 h. As shown in Figure 2(a), the presence of MacCM increased PAI-1 mRNA in SGBS adipocytes; already 5% MacCM induced PAI-1 mRNA by about 2-fold. In line with these findings, treatment of primary human *ex vivo* differentiated adipocytes obtained from healthy donors with MacCM increasing PAI-1 mRNA by about 1.6-fold (Figure 2(b)). Thus, these data suggested that obesity mimicking inflammatory conditions lead to an upregulation of PAI-1.

### 3.3. Resveratrol Reduces Upregulation of PAI-1 Gene Expression in an *In Vitro* Model of Inflamed Human Adipose Tissue

**Figure 3:**
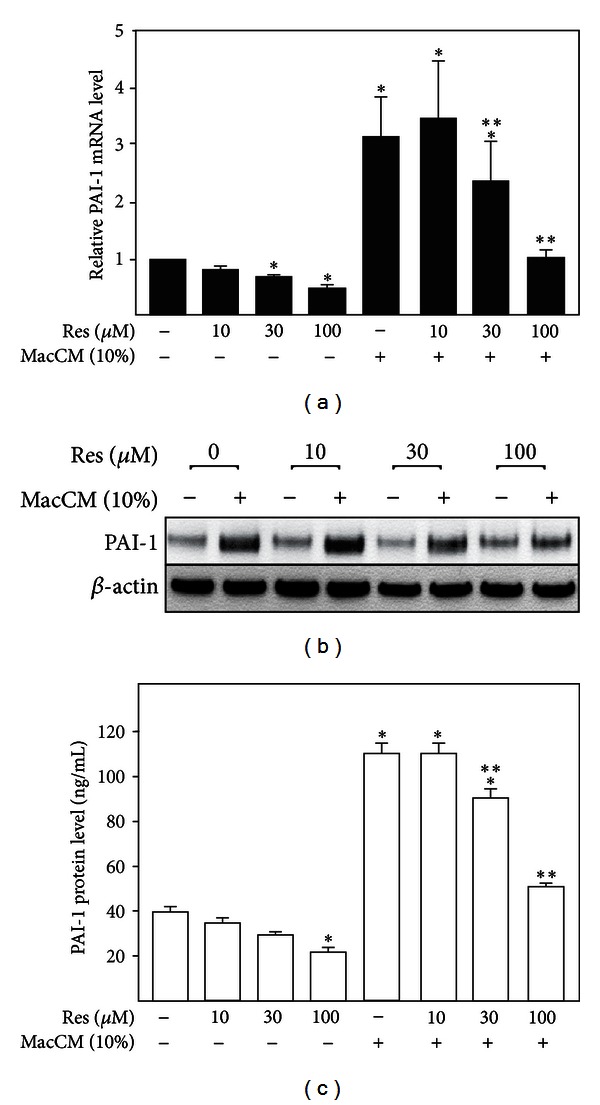
Resveratrol abolished the MacCM-dependent PAI-1 induction in SGBS adipocytes. SGBS adipocytes were treated with the indicated doses of resveratrol (Res), 10% MacCM, or a combination of Res and 10% MacCM for 48 h. (a) PAI-1 mRNA levels were analyzed by qPCR and results were normalized to HPRT. *Significant difference untreated versus Res or MacCM, **significant difference MacCM treated versus MacCM + Res. (b) Total cell protein lysates were isolated and subjected to Western blot analysis using an antibody against PAI-1 and *β*-actin as a loading control. (c) Accumulation of PAI-1 protein in media was measured by ELISA. *Significant difference untreated versus Res or MacCM, **Significant difference MacCM treated versus MacCM + Res.

To determine the effect of resveratrol on the elevated PAI-1 mRNA and protein levels under inflammatory conditions, SGBS adipocytes were cultured in the absence or presence of different concentrations of resveratrol, 10% MacCM, or a combination of both for 48 h. Treatment of SGBS adipocytes with increasing doses of resveratrol alone resulted in a concentration-dependent reduction of PAI-1 mRNA and protein levels (Figures 3(a), 3(b) and 3(c)). Incubation of cells with MacCM induced PAI-1 mRNA levels and PAI-1 protein levels by about 3-fold (Figures 3(a), 3(b) and 3(c)). The MacCM-dependent induction of PAI-1 mRNA and protein levels was abolished in the presence of 100 *μ*M resveratrol (Figures 3(a), 3(b) and 3(c)). Together, these data suggested that PAI-1 gene expression is enhanced under inflammatory conditions and that this induction is antagonized by the action of resveratrol. 

### 3.4. The Effects of Resveratrol on PAI-1 Expression Are Not Mediated via Sirt1, AMPK, or PI3K

Resveratrol has been shown to modulate several key signaling molecules in adipocytes, including Sirt1 [50, 51], AMPK [52–54], and PI3K/Akt [52, 55–57]. To examine whether Sirt1, AMPK, and/or PI3K are involved in the resveratrol-dependent downregulation of PAI-1 gene expression, we used specific inhibitors of these signaling pathways as well as knockout cells. Concerning the inhibitor studies, SGBS adipocytes were incubated with DMSO as a vehicle control, the Sirt1 inhibitor sirtinol or PI3K inhibitor LY294002 along with resveratrol, MacCM, or combinations, and the PAI-1 mRNA levels were determined 48 h after treatment. 

**Figure 4:**
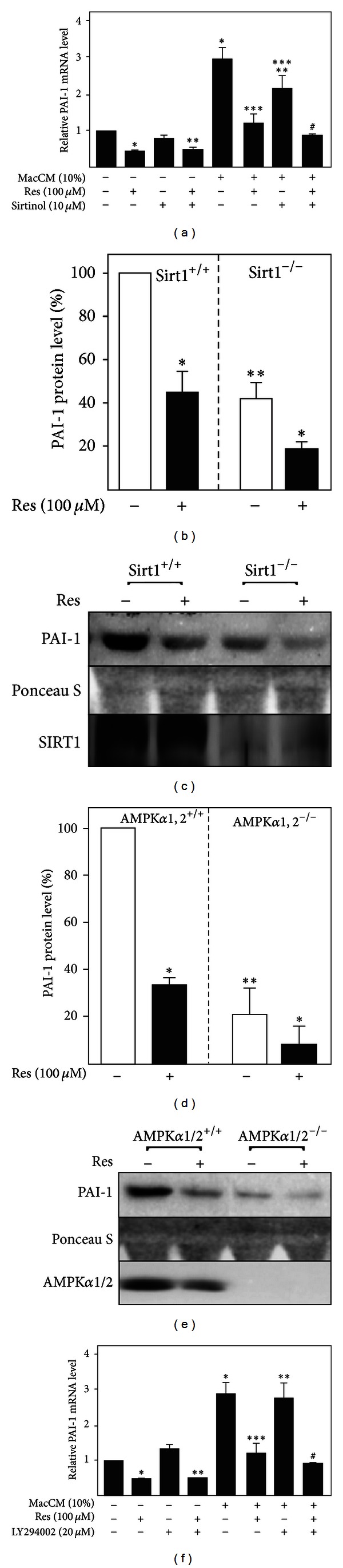
The effects of resveratrol on PAI-1 gene expression in SGBS adipocytes are not mediated via SIRT1, AMPK and PI3K. (a) Where indicated SGBS adipocytes were treated with 10 *μ*M sirtinol, resveratrol (Res, 100 *μ*M), and MacCM (10%) for 48 h. (a) PAI-1 mRNA levels were analyzed with qPCR and results were normalized to HPRT. *significant difference untreated versus Res, sirtinol, or MacCM; **significant difference untreated versus Res + sirtinol or Res + MacCM; ***significant difference MacCM versus MacCM + Res or MacCM + sirtinol; ^#^significant difference MacCM versus MacCM + Res + sirtinol. (b) SIRT1^+/+^ and SIRT1^−/−^ mouse embryonic fibroblasts were treated with 100 *μ*M resveratrol (Res) or vehicle control (DMSO) for 24 h. The PAI-1 and SIRT1 protein levels were measured by Western blot. *Significant difference untreated versus Res, **significant difference wild-type versus knockout cells. (c) Representative Western blot. (d) AMPK*α*1/2^+/+^ and AMPK*α*1/2^−/−^ mouse embryonic fibroblasts were treated with 100 *μ*M resveratrol (Res) or vehicle control (DMSO) for 24 h. The PAI-1 and AMPK*α*1/2 protein levels were measured by Western blot. *significant difference untreated versus Res, **significant difference wild type versus knockout cells. (e) Representative Western blot. (f) Where indicated SGBS adipocytes were treated with 20 *μ*M LY294002, 100 *μ*M resveratrol (Res), and 10% MacCM for 48 h. The PAI-1 mRNA levels were measured by qPCR and results were normalized to HPRT. *Significant difference untreated versus Res, LY294002, or MacCM; **significant difference untreated versus Res + LY294002 or Res + MacCM; ***significant difference MacCM versus MacCM + Res or MacCM + LY204002; ^#^significant difference MacCM versus MacCM + Res + LY294002.

In line with the above mentioned results, resveratrol decreased PAI-1 mRNA levels in SGBS adipocytes cultured either with or without MacCM. Sirtinol treatment alone slightly reduced the basal expression of PAI-1 mRNA in SGBS adipocytes (Figure 4(a)). However, sirtinol had no significant effect on the resveratrol-dependent downregulation of the PAI-1 mRNA in SGBS adipocytes incubated with MacCM (Figure 4(a)). To further rule out the role of Sirt1 in resveratrol-dependent regulation of PAI-1 expression, we examined the effect of resveratrol on the PAI-1 protein levels in Sirt1-deficient (Sirt1^−/−^) mouse embryonic fibroblasts (MEFs). Although the basal PAI-1 protein levels were lower in Sirt1^−/−^ MEFs, resveratrol treatment decreased the PAI-1 levels in both wild-type (Sirt1^+/+^) and the Sirt1^−/−^ MEFs by about 50% (Figures 4(b) and 4(c)). Together, these results indicate that although Sirt1 *per se* might be involved in the regulation of PAI-1 gene expression, the resveratrol-dependent modulation of PAI-1 gene expression is independent of Sirt1. 

Next we investigated the role of AMPK in the resveratrol-dependent regulation of PAI-1 expression. For this purpose we used wild-type AMPK*α*1/2^+/+^ (AMPK*β*1/2^+/+^) and AMPK*α*1/2-deficient (AMPK*α*1/2^−/−^) MEFs and measured PAI-1 protein levels after treatment with resveratrol by Western blot. The basal PAI-1 protein levels were significantly lower in AMPK*α*1/2^−/−^ MEFs, but again resveratrol treatment resulted in a significant decrease (by about 65%) of the PAI-1 protein levels in both wild-type and the AMPK*α*1/2^−/−^ MEFs (Figures 4(d) and 4(e)). Together, these data show that even though AMPK itself might be involved in the regulation of PAI-1 gene expression, the resveratrol-dependent downregulation of PAI-1 is mediated by an AMPK-independent mechanism. 

We further studied whether the PI3K/Akt pathway is involved in resveratrol-dependent downregulation of PAI-1 and used the PI3K inhibitor LY294002. While resveratrol treatment reduced PAI-1 mRNA levels by about 50% in both untreated and MacCM-treated SGBS adipocytes, LY294002 treatment did not change the basal PAI-1 mRNA levels (Figure 4(f)). Furthermore, incubation with LY294002 did not block the decline of PAI-1 mRNA levels by resveratrol in both untreated and MacCM-treated SGBS adipocytes, implicating that the PI3K/Akt pathway is not involved in the resveratrol-modulated downregulation of PAI-1. 

### 3.5. ROS Formation and the Antioxidant Transcription Factor Nrf2 Do Not Contribute to the Effects of Resveratrol on PAI-1 Gene Expression

**Figure 5:**
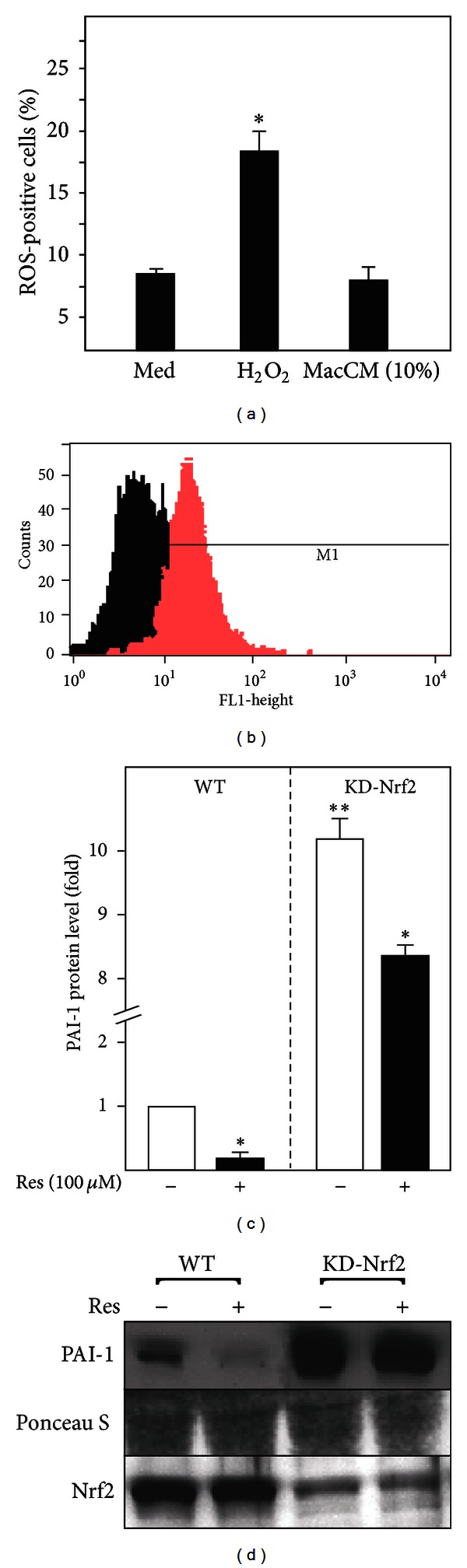
Macrophage-conditioned media do not induce ROS formation in human adipocytes and the antioxidant transcription factor Nrf2 does not contribute to the resveratrol effects on PAI-1 gene expression. (a), (b) SGBS adipocytes were labelled with 2.5 *μ*M CM-H_2_DCFDA and then treated with 50 *μ*M H_2_O_2_ and 10% MacCM for 15 min. ROS production was analyzed by flow cytometry. (a) ROS-positive adipocytes after treatment with H_2_O_2_ and MacCM; *significant difference untreated versus H_2_O_2_. (b) Histograms of ROS-positive cell percentage in cells cultured in medium or treated with H_2_O_2_ for 15 min. (c) Nrf2^+/+^ and Nrf2 knock-down mouse embryonic fibroblasts were treated with 100 *μ*M resveratrol (Res) or corresponding vehicle control (DMSO) for 24 h. The PAI-1 and Nrf2 protein levels were measured by Western blot. *Significant difference untreated versus Res, **significant difference wild type versus knockout cells. (d) Representative Western blot.

Obesity and inflammation are associated with increased ROS formation [58, 59] and ROS-mediated signaling has been reported to regulate PAI-1 gene expression [60, 61]. Resveratrol is well known for its antioxidant potential and therefore we aimed to determine whether the observed MacCM-dependent induction of PAI-1 gene expression and hence the effects of resveratrol were dependent on ROS generation. To address this issue, ROS levels were examined in SGBS adipocytes treated with MacCM or for the purpose of a positive control with H_2_O_2_. Intracellular ROS levels increased upon treatment with H_2_O_2_. By contrast, no changes in ROS generation were detected in MacCM-treated SGBS adipocytes (Figures 5(a) and 5(b)) implying that MacCM-dependent PAI-1 induction is independent of ROS. 

The NFE2-related factor 2 (Nrf2) is a key transcription factor, involved in the primary cellular defense against the cytotoxic effects of oxidative stress [62]. To further exclude the possibility that the effects of MacCM and resveratrol on PAI-1 gene expression are independent of ROS, we used wild-type and Nrf2 knockdown MEFs. Interestingly, the knockdown of Nrf2 increased PAI-1 protein levels compared to the wild-type cells (Figures 5(c) and 5(d)) but the addition of resveratrol caused a decrease in PAI-1 levels by about 80% in wild-type cells and by about 35% in Nrf2 knockdown cells (Figures 5(c) and 5(d)). Thus, the antioxidant transcription factor Nrf2 is not involved in mediating the resveratrol effects on PAI-1 expression. 

### 3.6. The Effects of Resveratrol on PAI-1 Gene Expression in an* In Vitro* Model of Inflamed Adipose Tissue Are NF*κ*B Dependent

**Figure 6:**
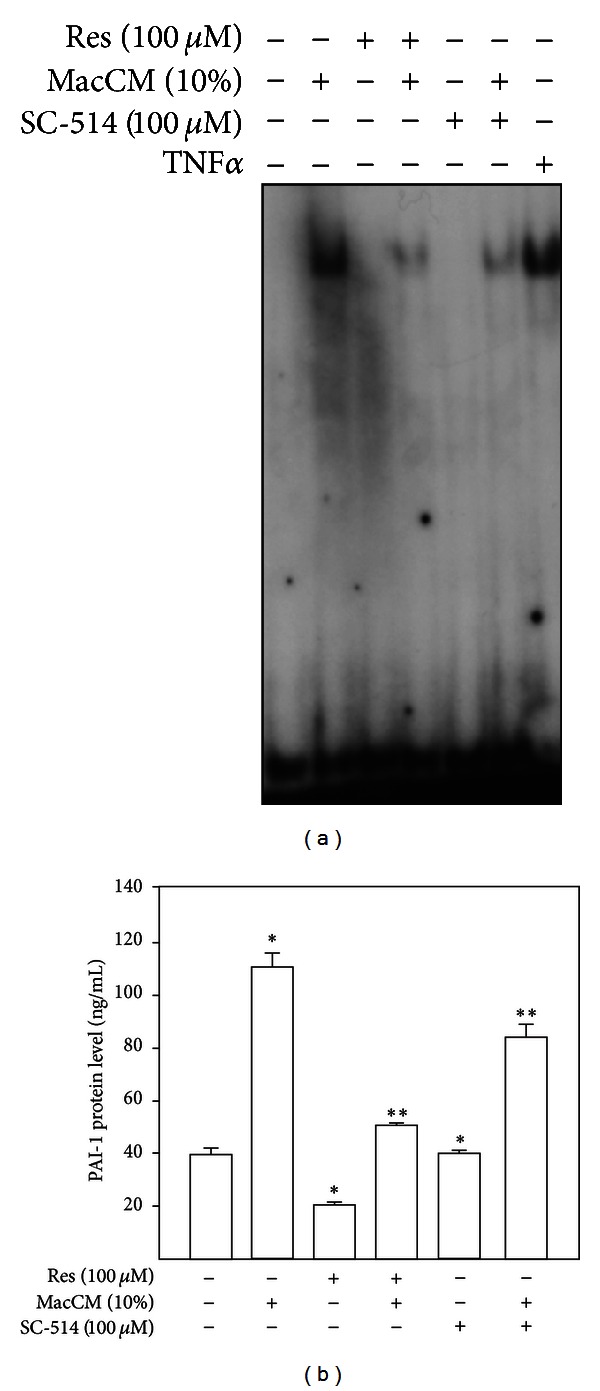
Resveratrol-mediated suppression of NF*κ*B DNA binding activity did not abrogate PAI-1 gene expression. (a) Electrophoretic mobility shift assay using a 5′-end-labelled consensus oligonucleotide for NF*κ*B binding and nuclear extracts from SGBS adipocytes. SGBS adipocytes were treated with 10% MacCM, 100 *μ*M resveratrol, 100 *μ*M SC-514, or combination of them and then incubated for 1 h. The DNA-protein complexes were separated by electrophoresis on 5% native polyacrilamide gels and visualized by phosphoimaging. (b) SGBS adipocytes were treated with 10% MacCM, 100 *μ*M resveratrol (Res), 100 *μ*M SC-514, or a combination of both Res and 10% MacCM or SC-514 and MacCM for 48 h. Accumulation of PAI-1 protein in media was measured by ELISA. *Significant difference untreated versus MacCM, Res or SC-514; **significant difference MacCM treated versus MacCM + Res or MacCM + SC-514.

The reduction of PAI-1 expression by resveratrol under inflammatory conditions may be partially explained by the ability of resveratrol to suppress the activity of NF*κ*B, a transcription factor critically involved in inflammation. Therefore, we examined the effect of resveratrol on the DNA-binding activity of NF*κ*B in the model of inflamed adipose tissue. By performing EMSA, we found that an oligonucleotide with a NF*κ*B binding site was able to form a single DNA-protein complex (Figure 6(a)) when incubated with nuclear extracts from SGBS adipocytes treated with either MacCM or the established NF*κ*B activator, TNF-*α*. The NF*κ*B DNA-binding activity was significantly reduced in nuclear extracts from cells treated with MacCM and resveratrol or MacCM and SC-514 (Figure 6(a)). These data demonstrate that resveratrol can lead to a suppression of NF*κ*B DNA-binding activity under inflammatory conditions in SGBS adipocytes. Based on the above findings, demonstrating the suppressive effect of resveratrol on NF*κ*B DNA-binding, we expected that inhibition of NF*κ*B by resveratrol would reduce PAI-1 gene expression. Accordingly, SGBS adipocytes were treated with MacCM, resveratrol, and SC-514 alone or in combination, and PAI-1 protein levels were measured by ELISA. In line, resveratrol and SC-514 reduced MacCM-dependent PAI-1 protein induction (Figure 6(b)), though the effects of resveratrol were much more pronounced than the effects of the NF*κ*B inhibitor SC-514 alone. These data strongly suggest that the effects of resveratrol on PAI-1 gene expression in SGBS adipocytes are NF*κ*B dependent. 

## 4. Discussion

In this study we investigated the human PAI-1 expression in response to resveratrol in human SGBS adipocytes and in a model of inflamed human adipose tissue. Our data demonstrated several new findings with respect to resveratrol and human PAI-1 regulation under obesity-mimicking conditions. First, it was found that resveratrol downregulated PAI-1 mRNA and protein levels in a time- and concentration-dependent manner in human SGBS adipocytes. Second, the inhibitory effect of resveratrol on PAI-1 was even stronger on the obesity-associated and inflammation-dependent induction of PAI-1. Third, while resveratrol exerted its effects on inflammatory-dependent PAI-1 gene expression mainly via inhibition of NF*κ*B, signaling via Sirt1, AMPK, PI3K, ROS, and Nrf2 did not mediate the effect of resveratrol on PAI-1 production. 

Obesity represents a risk factor for the development of diseases like type 2 diabetes, hypertension, atherosclerosis and myocardial infarction. Intriguingly, obesity is also associated with a state of chronic low-grade inflammation characterized by elevated plasma concentrations of proinflammatory cytokines (IL-6, IL-1 and TNF*α*), chemokines (monocyte chemotactic protein 1, MCP-1), and adipokines (haptoglobin, PAI-1, leptin, visfatin, resistin and VEGF) [63]. Plasma PAI-1 levels are considerably enhanced in obese humans and in patients with insulin resistance, type 2 diabetes, and cardiovascular diseases [23, 64]. The adipose tissue appears to be the major source of elevated PAI-1 levels observed in obesity [65, 66] maybe as a result of its increased capacity to produce PAI-1 and/or as an effect of direct stimulation of adipocytes by hormones and cytokines upregulated in obesity [67]. Resveratrol is capable of attenuating obesity-associated inflammatory responses by inducing changes in the secretion profile of adipocytes [68–71]. In particular resveratrol inhibited TNF*α*-dependent PAI-1 upregulation in 3T3-L1 adipocytes [68, 70], IL1*β*-stimulated PAI-1 secretion [69], and PAI-1 production in human SGBS adipocytes [71]. These data are very much in line with the results from the present study where we have shown that resveratrol not only downregulated PAI-1 expression (Figure 1) but even exerted a stronger effect on PAI-1 in a model of inflamed human adipose tissue (Figures 2 and 3). Although all these data indicate that resveratrol can alleviate obesity-induced upregulation of PAI-1 in adipose tissue, it has not been fully elucidated by which molecular mechanisms resveratrol exerts its effect on PAI-1 under inflammatory conditions. 

Calorie restriction is considered to be one of the most effective nutritional interventions protecting against obesity, diabetes, and cardiovascular disease [72]. The obesity-related enhancement of PAI-1 levels also appeared to be reversible by calorie restriction diet or calorie restriction mimetics [26, 27]. Several signaling pathways have been implicated in mediating the calorie restriction effect—the sirtuin pathway, the adenosine monophosphate (AMP) activated protein kinase (AMPK) pathway, and the insulin-like growth factor (IGF-1)/insulin signaling pathway (as discussed by [73]). In rodents calorie restriction and calorie restriction mimetics seem to extend the life span and are linked to silent mating type information regulation 2 homolog 1 (Sirt1) activation (references in [74]). Resveratrol was identified as a Sirt1 activator [75] and gained interest in a number of pathological settings—among them obesity. In line, the anti-inflammatory effects of resveratrol in adipocytes as well as in human adipose tissue were shown to be mainly dependent on Sirt1 activation [69, 76, 77]. However, in our study, neither inhibition of Sirt1 with sirtinol nor deficiency of Sirt1 was able to abrogate the resveratrol effects on PAI-1 (Figures 4(a), 4(b) and 4(c)) implicating that Sirt1 activation is not necessary to mediate the action of resveratrol on PAI-1 synthesis under inflammatory conditions.

Resveratrol is known to exert pleiotropic effects on cells and Sirt1 activation is not the only effect via which resveratrol exerts its beneficial actions on obesity-associated pathological consequences [29, 30, 78]. Therefore, the inhibitory effect of resveratrol on PAI-1 production in obesity may result from modulation of different signaling pathways. Some of the beneficial effects of resveratrol against diet-induced obesity and insulin resistance were mediated via AMPK activation [29, 35, 36, 38, 78, 79]. In addition, increasing evidence suggests that AMPK has anti-inflammatory actions [80, 81]. Therefore, we have tested whether the effects of resveratrol on PAI-1 expression are mediated via AMPK. Our results demonstrated that resveratrol-dependent downregulation of PAI-1 was still preserved in AMPK-deficient cells (Figures 4(d) and 4(e)) pointing out that resveratrol acts on PAI-1 in an AMPK-independent mechanism. 

A number of experimental observations have demonstrated that the PI3K/Akt pathway represents an important signaling cascade in the initiation of the inflammatory response. Although we showed in an earlier study that resveratrol inhibits PI3K-driven Akt phosphorylation in SGBS cells [55] the PI3K inhibitor, LY294002, could not abrogate the resveratrol-dependent downregulation of PAI-1 (Figure 4(f)) implicating that the PI3K/Akt pathway is also not involved in the modulation of PAI-1 expression by resveratrol. 

Inflammation is well known to exist in combination with oxidative stress which in turn is a potent modulator of PAI-1 gene expression in different systems [60, 82] as well as in this study. In this context, an important transcription factor mediating responses to oxidative stress is Nrf-2 [83]. Resveratrol supplementation has been shown significantly to increase Nrf2 activity in humans after a meal [84]. However, the conditions of our inflammatory model did not induce ROS generation (Figures 5(a) and 5(b)). In line with that, the knockdown of Nrf2 did not impair the resveratrol effect on PAI-1 secretion (Figures 5(c) and 5(d)). 

An increase in plasma PAI-1 levels observed in obesity can also be the result of a cytokine-dependent induction of PAI-1 transcription where the proinflammatory cytokines such as IL-1, IL-6, and TNF*α* play the major role [85–87]. Interestingly, no STAT3 binding element participating in the IL-6 response could be mapped in the PAI-1 promoter whereas the so-called NF*κ*B-like sites within the PAI-1 promoter and a TNF*α*-responsive enhancer located 15 kb upstream of the transcription start site were shown to participate in response to IL-1 and TNF*α* (references in [14]). 

Nuclear factor (NF)*κ*B is a transcription factor with a central role in the induction of a chronic inflammatory state associated with obesity, development of type 2 diabetes, cardiovascular risk, and insulin resistance [88]. Previous reports established resveratrol as an inhibitor of NF*κ*B [41, 89] and resveratrol treatment of TNF*α*-stimulated adipocytes reduced the expression of proinflammatory cytokines [88]. Therefore, our results showing that the resveratrol effects on PAI-1 gene expression were NF*κ*B-dependent (Figure 6) are in line with those findings. 

Interestingly a number of *in vivo* and *in vitro* studies showed an inhibitory role of the resveratrol target Sirt1 on NF*κ*B signaling [76, 77, 90, 91]. Similarly, AMPK signaling has been shown to inhibit the inflammatory responses induced by NF*κ*B via several downstream targets of AMPK (references in [92]). Moreover, several previous findings have demonstrated that the PI3K/Akt pathway has a crucial role in the activation of the NF*κ*B pathway [93, 94]. Based on these studies and the role of resveratrol as a Sirt and AMPK activator, PI3K inhibitor as well as ROS scavenger, and we were expecting that Sirt, AMPK, PI3K, or ROS would be involved in the resveratrol effects. Surprisingly, none of these upstream NF*κ*B modulators contributed to the effects of resveratrol; however, in line with previous studies [77, 88, 95–100] our findings show that resveratrol can act as an NF*κ*B inhibitor, most likely via so far not a characterized pathway. 

## 5. Conclusions

Together, our study showing that resveratrol mediates an inhibitory effect on PAI-1 may be useful to further establish PAI-1 as a marker for obesity-associated inflammatory conditions. In addition, we add at least one novel aspect to the pleiotropy of the resveratrol action by showing that it can act as an NF*κ*B inhibitor without involving Sirt1, AMPK, PI3K or ROS.
